# Analysis of Cytoplasmic and Secreted Proteins of *Staphylococcus aureus* Revealed Adaptive Metabolic Homeostasis in Response to Changes in the Environmental Conditions Representative of the Human Wound Site

**DOI:** 10.3390/microorganisms8071082

**Published:** 2020-07-20

**Authors:** Mousa M. Alreshidi, R. Hugh Dunstan, Margaret M. Macdonald, Vineet K. Singh, Tim K. Roberts

**Affiliations:** 1 Department of Biology, College of Science, University of Ha’il, Hail P.O. 2440, Saudi Arabia; 2 Metabolic Research Group, Faculty of Science, School of Environmental and Life Sciences, University Drive, Callaghan, NSW 2308, Australia; hugh.dunstan@newcastle.edu.au (R.H.D.); margaret.macdonald@newcastle.edu.au (M.M.M.); tim.roberts@newcastle.edu.au (T.K.R.); 3 Microbiology and Immunology, Kirksville College of Osteopathic Medicine, A.T. Still University of Health Sciences, 800 West Jefferson Street, Kirksville, MO 63501, USA; vsingh@atsu.edu

**Keywords:** *S. aureus*, secreted proteins, cytoplasmic proteins, stress response

## Abstract

The pathogenesis of *Staphylococcus aureus* is mainly attributed to its capability to adjust to changes in environmental conditions, including those present on human skin or within a wound site. This study investigated the changes in the cytoplasmic and secreted proteins in *S. aureus* that occurred in response to alterations in the environmental parameters that could be found in the human wound site. In total, sixty differentially regulated cytoplasmic proteins were detected using a label-free quantification approach, and these proteins were classified into ten molecular functions: protein biosynthesis, glycolysis, signal transduction, metabolism, cell cycle, transport, energy generation, cell anchorage, nucleotide biosynthesis and unknown. These changes represented characteristic protein profiles when evaluated by principal component analysis. The bacterium responded to elevated NaCl at pH 6 by decreasing the abundance of the majority of cytoplasmic proteins, while at pH 8 there was an increase in the levels of cytoplasmic proteins in comparison to the untreated cells. The analysis of the secreted proteins showed that there was a high degree of difference in both the intensity and the distribution of many individual protein bands in response to environmental challenges. From these results, it was deduced that specific metabolic homeostasis occurred under each combination of defined environmental conditions.

## 1. Introduction

*Staphylococcus aureus* is a causative agent for many diseases worldwide, ranging from mild skin and tissue infections to highly severe infections, including toxic shock syndrome, endocarditis, and osteomyelitis [1]. *S. aureus* is considered the leading cause of nosocomial and community infections, although it is classified as a commensal bacterium. However, the exact mechanism that enables the switch from a commensal to a pathogenic lifestyle is unclear [2]. The threat of this bacterium lies in its remarkable and rapid capacity to sense environmental signals and modulate the cellular responses; this is the major challenge for clinicians and scientists [3,4,5]. The capacity of *S. aureus* to adapt to fluctuations in environmental conditions was proposed to be due to its ability to induce considerable changes in cytoplasmic metabolites and proteins [4,6,7]. These changes usually result in extraordinary alterations in the external morphology. e.g., cell size, cell wall thickness and ultimately colony size and type [8,9]. Small colony variants (SCVs) represent a good model of an altered bacterial phenotype where the colonies formed are ten-fold smaller in diameter compared with normal colonies [10,11,12]. These variants are highly associated with persistent and recurrent infections, although they display attenuated virulence factors [11,13]. Previous studies have demonstrated that *S. aureus* colonies can be formed into SCVs after growth in less favorable conditions, including low temperature, low pH, and antibiotic exposure [8,9,12,13]. Thus, the formation of SCVs is possibly a mechanism used by staphylococcal species to combat adverse conditions, but the exact mechanism that enables the switch from a normal colony to a more resilient phenotype, such as a SCV, is unknown.

The human wound site represents a major potential avenue for pathogen invasion and colonization [14]. In fact, *S. aureus* is the most frequently isolated bacterium from wound infections even though the environmental conditions in the wound site are diverse and dynamic during the healing process [15]. Therefore, understanding this bacterial survival in a wound site becomes essential to developing new antimicrobial strategies. These conditions include a variable pH, osmotic pressure, and temperature. The wound pH can be acidic or neutral but it becomes basic during the first stage of the healing process [16]. The temperature varies from 35 °C to 39 °C and osmolality fluctuates from 275–300 milliosmoles/L [17,18]. Several studies have investigated how *S. aureus* and *Staphylococcus lugdunensis* adapt to changes in the environmental conditions, mimicking wound site conditions: pH 6–8, temperature 35–39 °C, and adding NaCl to increase osmolality by up to 5%. Staphylococcal species adapted to these conditions by inducing considerable alterations in the composition of membrane fatty acids and cytoplasmic amino acids, as well as changes in ribosomal proteins and cell sizes [6,19,20]. The adaptive changes also demonstrated in *S. aureus* under a similar design were consistent with the formation of metabolic phenotypes characteristic of the prevailing environmental conditions [21]. These results support the hypothesis that *S. aureus* can acclimatize to changes in the environmental conditions by inducing major changes in cellular composition and external morphology. Therefore, the current study used the same experimental design to investigate the responses of the cytoplasmic and secreted proteins after growth in the conditions representative of the skin surface and wound site. It was hypothesized that major changes in cytoplasmic and secreted proteins would occur as a part of adaptation processes to alterations in environmental conditions.

## 2. Experimental Procedures

### 2.1. Bacterial Strain and Growth Conditions

The bacterial strain used in this study was a clinical isolate of *S. aureus* from patients that had been suffering from chronic muscle pain [22]. This isolate has been used in subsequent investigations to investigate metabolic responses to environmental stresses [8,9,23]. The isolate has been maintained as culture stock on horse blood agar (HBA), and preserved appropriately on sterile glass beads at −80°C with a regular sub-culturing to maintain viability [24]. The identity of the isolate was checked regularly using API^TM^ Staph biochemistry (BioMérieux, Australia, Pty Ltd) and through polymerase chain reaction PCR [25].

### 2.2. Experimental Design and Statistical Rational

For the analysis of cytoplasmic proteins, bacterial cells were grown in the same conditions described in [6] as part of a larger experiment created by the MODDE software (9.0, Umetrics, Umeå, Sweden) [19]. This allowed the establishment of a multifactorial design, optimized to assess the impact of varying three factors at a time, which in this case were temperature, pH and osmotic pressure. Selected environmental conditions were chosen for the analyses of both cytoplasmic and secreted proteins. In all cases, *n* = 4. The reference control (A) included the cells grown under the ideal conditions of pH 7 at 37 °C, with no added NaCl in the tryptic soy broth medium (TSB); a “centroid” set of samples represented the mid-range conditions of the larger experimental design [19] with the conditions of pH 7 at 37 °C with 2.5% NaCl added in TSB (B); four sets of experimental conditions were applied with 1) 35 °C and pH6 with no added NaCl in TSB (C); 2) 35 °C and pH6 with NaCl added (5%) in TSB (D); 3) 35 °C and pH8 with no added NaCl in TSB (E); and 4) 35 °C and pH8 with NaCl added (5%) in TSB (F). The normal range of osmolality for plasma is 275–300 milliosmoles/L, the osmolarity of the TSB was measured as 300 mosmoles L^−1^ and the calculated osmolarity of the PBS was 306 mosmoles L^−1^ [26]. The sodium chloride ranged from 0 to an additional 5% loading on the TSB, taking the final concentration well beyond the plasma concentration which may occur on skin surfaces with the added electrolytes from the natural moisturizing factor and sweat [27] and has also been noted in wound site responses to traumatization [16]. The temperature range was 35–37 °C and the pH ranged from 6 to 8. An overnight starter culture (50 mL) of *S. aureus* was grown for 16 h in Tryptic Soy Broth (TSB) at 37 °C with constant agitation (120 rpm), to be used as an inoculum for the growth experiments. Replicates of each condition containing 95 mL TSB culture media were inoculated with 5 mL of overnight culture in 500 mL conical flasks, which were then grown until mid-exponential phase with constant agitation (120 rpm).

For the analysis of secreted proteins, the cells were grown to a stationary phase under a subset of environmental conditions including a reference control with cells grown under ideal conditions at pH 7, 37 °C and with no added NaCl in a tryptic soy broth medium (TSB). A “centroid” set of samples represented the mid-range conditions of the larger experimental design as described in [19] with conditions at pH 7, 37 °C and with NaCl added (2.5%) in the TSB. An experimental set of conditions was applied at 39 °C and pH 6 with NaCl added (5%) to the TSB.

The obtained data were analyzed using an analysis of variance; Duncan’s test was used to determine the significance of all the proteins in all experimental groups in comparison to the reference control. Principle component analysis (PCA) and a hierarchical heatmap used log transformed data by R statistical software. For the PCA, built-in packages were used, and the data were subjected to mean centering prior to the PCA calculations. The heatmap was generated using the g plots package. Two and three-dimensional PCA plots were generated by PCA3D package. The Venn diagram was designed using the InteractiVenn website [28]. Gene ontology functional enrichment analysis by the PANTHER classification system was applied to determine the putative function proteins [29].

### 2.3. Extraction and Estimation of Cytoplasmic Proteins

The proteins were extracted from the cytoplasm of dried cells from both the reference control and the treated samples. The cells were resuspended in 500 µL of SDS Lysis Buffer containing 2% SDS, 0.375 M Tris pH 6.8, 3.4 M sucrose (Sigma-Aldrich, St. Louis, MO, USA) and one tablet of protease inhibitor (complete Mini, Roche Diagnostics), thoroughly mixed and heated at 100 °C for 6 min. Cell debris was removed by centrifugation at 14,000 rpm for 25 min. The supernatant containing the extracted proteins was carefully removed and stored at –20 °C until further investigation. Protein concentrations in each sample were determined using the Bicinchoninic Acid (BCA^TM^)assay (Bio-Rad, Hercules, CA, USA) following the manufacturer’s instructions, and Bovine Serum Albumin (BSA) was used as the reference standard.

### 2.4. Sodium Dodecyl Sulphate Polyacrylamide Gel Electrophoresis (SDS-PAGE) and Staining Procedures

SDS-PAGE gels were made according to these procedures: 4% stack gel and 12% resolving gel (30% Acrylamide, 0.5 M Tris-HCl, pH 6.8). Silver staining was performed according to the method used by [30]. Briefly, the procedure involved a gel fixation wash three times using 5% acetic acid and 10% ethanol, and subsequently washes in triplicate using 10% ethanol. The gels were then rinsed three times with distilled water and incubated in 2.9 µg of sodium thiosulphate for 2 min followed by three washes with distilled water. The gel was silver stained for 5 min, and quickly rinsed four times with distilled water before incubation in development solution (sodium bicarbonate, sodium thiosulfate and formaldehyde) till the appearance of the protein bands. The reaction was inactivated by adding 1% of acetic acid to the gel.

### 2.5. Protein Reduction, Alkylation and Digestion

One hundred and fifty micrograms of proteins from both the reference control and treated samples was precipitated using sample/methanol/chloroform in the ratio of 1:1:0.5 (v/v/v). The mixture was vortexed and centrifuged at 14,000 rpm for 15 min. The upper layer was discarded and 75% of the original volume of methanol was added to each replicate and centrifuged at 14,000 rpm for 20 min. The supernatant was removed without disrupting the protein pellet and was air dried for 10 min. The protein pellet was washed two times with 500 µL of cold acetone (kept at −20 °C). The precipitated proteins were resuspended with 150 µL of ammonium bicarbonate (25 mM) and 10 mM dithiothreitol (DTT) (final concentration) and boiled at 95 °C for 10 min. The samples were cooled for 5 min and subsequently 55 mM of iodacetamide was added, the sample was vortexed and kept in the dark at room temperature for 35 min. The protein was digested using trypsin enzyme (Promega sequence grade, Madison, Wisconsin, USA) in a ratio of 50:1 (protein: trypsin) in 25 mM ammonium bicarbonate, and the sample incubated overnight at 37 °C with constant shaking. The next morning, the trypsin was inactivated by adding 0.1% (v/v) formic acid. The samples were then spun at 14,000 rpm for 40 min to remove the undigested proteins.

### 2.6. LC–MS/MS Analysis of Cytoplasmic Proteins

The peptides yielded from the tryptic digestion of the cytoplasmic proteins were analyzed by nanoLC–MS/MS using a LTQ Orbitrap Elite mass spectrometer (Thermo Scientific, Thermo Fisher, Waltham, MA, USA) coupled to an Ultimate 3000 RSLC nanosystem (Dionex, Idstein, Germany). The nanoLC system was equipped with an Acclaim Pepmap nano-trap column (Dionex –C18, 100 Å, 75 µm × 2 cm) and a Thermo EASY-Spray column (Pepmap RSLC C18, 2 µm, 100 Å, 75 µm × 25 cm). Four µL of the peptide mix was loaded onto the enrichment (trap) column at an isocratic flow of 5 µl/min of 3% CH_3_CN, containing 0.1% formic acid for 5 min before the enrichment column was switched in-line with the analytical column. The eluents used for the liquid chromatography were 0.1% (v/v) formic acid (solvent A) and 100% CH_3_CN/0.1% formic acid (v/v). 0.1% formic acid (v/v). The following flow gradient was used: 3% to 6% B for 1 min, 6% to 10% B in 12 min, 10% to 30% B in 20 min, 30% to 45% B in 2min, 45% to 80% B in 2 min and maintained at 80% B for 3 min followed by equilibration at 3% B for 7 min before the next sample injection. The LTQ Orbitrap Elite mass spectrometer was operated in the data-dependent mode with a nano ESI spray voltage of +2.0 kv, a capillary temperature of 250 °C and an S-lens RF value of 60%. 

A data-dependent mode was used, whereby the spectra were acquired first in positive mode with full scan scanning from m/z 300 to 1650 in the FT mode at 120,000 resolution, followed by Collision induced dissociation (CID) in the linear ion trap, with the ten most intense peptide ions with charge states ≥2 isolated and fragmented using a normalized collision energy of 35 and the activation Q of 0.25.

### 2.7. Database Search and Protein Quantification

The mass spectra were searched using Mascot 2.3 (Matrix Science, London, UK) as part of the Proteome Discoverer 1.4 Workflow (Thermo Scientific) against the Uniprot database (with 26,167,536 sequences at the time of search) and the Firmicutes taxonomy. The search parameters used were: fixed modification (carbamylation of cysteine C; 57), variable modification (oxidation of methionine M; 16), two missed tryptic cleavages, 10 ppm peptide mass tolerance and 0.6 Da fragment ion mass tolerance. The false-discovery rate (derived from corresponding decoy database search) was less than 1%.

The MS result files were imported to SIEVE (Version2.1 Thermo Scientific, San Jose, CA, USA) for the label-free relative quantification of the peptides between the reference control and the treated samples. The experiment workflow was selected as the “Control Compare Trend Analysis” which allows comparing the control samples to more than one treated sample. The three main steps include: the alignment of all peptides, which considers the reproducibility of the replicates and the correlation of all files to the reference file. The second step was to create the frames (Peptides) and the following parameters were formatted to create the frames (10,000 frames, with signal threshold > 125,000, m/z which starts at 350 and stops at 1,300, and a retention time (RT) which starts = 10 and stops = 60 min) [31]. The third step was the identification of protein and this was done by importing the Mascot 2.1 result to SIEVE 2.1. A filer was applied to the frames table and subsequently the peptides table. The Frame data generated by SEIVE was exported to an Excel^®^ (Microsoft^®^, Redmond, Washington, USA) spreadsheet file for further data analysis using ANOVA analysis, principal component analysis (PCA).

### 2.8. Analysis of Secreted Proteins

The cells were separated by centrifugation at 6000 *g* for 25 min and the supernatants were collected and filtered through 0.22 μm. The supernatants were precipitated by adding 100% of cold trichloroacetic acid (TCA) kept at (4 °C) to a final concentration of 15% and incubated overnight at 4 °C. The next day, the samples were centrifuged at 12,000 rpm for 30 min and washed four times with 96% cold ethanol kept at −20 °C to ensure the removal of TCA. The samples were air-dried, and each replicate was resolved in a 500 μL SDS-PAGE loading buffer SDS Lysis Buffer containing protein concentrations in each sample, which were determined using the BCA^TM^ assay (Bio-Rad) following the manufacturer’s instructions, and bovine serum albumin (BSA) was used as the reference standard. Fifteen micrograms of protein from each sample was run in SDS-PAGE to determine any large-scale differences in the protein production between the control and treated cells. The difference between the bands was determined by sight. Gel bands of interest were then cut and analyzed by LC–MS/MS. The excised bands were washed, reduced with DTT and alkylated with iodoacetamide followed by dehydration (as shown in the reduction alkylation). The gel bands were then rehydrated with 25 mM ammonium bicarbonate containing 70 ng Trypsin and incubated with gentle agitation overnight at room temperature. The samples were subsequently acidified (pH ~2) with a 10% trifluorocetaic acid (TFA) (v/v) solution to inactivate the trypsin, and the bands were sonicated (ultrasonic water bath) in increasing amounts of Acetonitrile (0%, 30% 50% 100%) for peptide extraction [30]. The samples containing the extracted peptides were then vacuum concentrated to remove the acetonitrile and resuspended in 15 uL LC loading buffer A (2% Acetonitrile, 0/1% TFA), spun at top speed for 5 min (to remove any particulates which can block the LC columns) before the supernatant was transferred to a Waters Autosampler vial.

The samples were analyzed by nanoflow reverse phase liquid chromatography (Dionex Ultimate 3000 RSLCnano, Dionex, Idstein, Germany) attached directly to an ESI 3D Ion Trap Mass Spectrometer (AmaZon ETD, Bruker GmbH, Preston, VIC, Australia) operating in MS/MS (CID) mode. Peptides were loaded at 5 uL/min onto a 5 um C18 nanoViper trap column (100 um × 2 cm, Acclaim PepMap100, Thermo) for desalting and pre-concentration. The peptide separation was then performed at 300 nl/min through an Acclaim nanoViper analytical column (2 um C18, 75 um × 15 cm) utilizing a gradient of 2–40% Buffer B (80% acetonitrile, 0.1% formic acid) over 60 min. The peptides were eluted directly into the nanoflow ESI Ion source of the MS system for the MS/MS analysis. The AmaZon Ion Trap system was set to perform the MS/MS on the top 5 ions present in each MS scan with an Ion exclusion time of 30 s. The raw MS files were then converted into a MASCOT Generic Format using DataAnalysis 4.1 and imported into the ProteinScape 2.1 platform (both Bruker, Bremen, Germany) for database searching. The searches were performed against the SwissProt (Bacterial taxonomy) and Uniprot (*S. aureus*) databases using an in-house licensed MASCOT server (version 2.3.02, Matrix Science). The number of allowed trypsin missed cleavages was set to 2. The carbamidomethylation of cysteine was set as a fixed modification, whereas the deamidation of asparagine and glutamine and the oxidation of methionine were set as the variable modifications. The parent ion tolerance was set to 1.2 Da with a fragment ion tolerance set to 0.7 Da. The peptide thresholds required a false positive rate of less than 0.05% with a low stringency Mascot score greater than 35. The spectra meeting these criteria were validated by manual inspection to ensure the accurate y- and b-ion detection with overlapping sequence coverage. The identity of the proteins was considered valid if it had at least five peptides and the mascot score was > 300.

## 3. Results

### 3.1. Cytoplasmic Proteomic Analysis

*S. aureus* (clinical strain) was cultured in tryptic soy broth to the mid-exponential phase of growth in multiple combinations of environmental conditions to mimic those on the human skin or within a wound site: pH 6–7, temperature 35–37 °C, and with 0–5% NaCl. The proteomic analysis revealed that sixty proteins were differentially regulated in one or more of the experimental groups. The protein quantification was conducted by considering the sum of all the peptide intensities per protein and at least two peptides were required to consider the protein quantification valid (the number of peptides for each protein is provided in the Supplementary Material (Table S1 and Table S2)). The profiles of the cytoplasmic proteins assessed in each of the cultures were consistent and reproducible within each replicate of the experimental groups, with certain compositional changes associated with each group (Table 1). Significant changes in the composition of cytoplasmic proteins were observed when the bacteria were grown in cultures with higher osmotic pressure (Group B, E and F). The bacterial cells responded to a high NaCl concentration at pH 8 by displaying higher abundances of most cytoplasmic proteins (F); in contrast, at pH 6 with high levels of NaCl (E), the cells showed lower abundances of proteins. indicating that at the higher NaCl concentrations, the pH had a powerful influence over the protein abundance. However, compared with the reference controls, when *S. aureus* was exposed to sets of conditions without additional NaCl, only a few changes occurred in the protein abundance (Table 1). For example, at pH 6 and 8 at 35 °C without NaCl (C and D), only nine and four proteins, respectively, were differentially regulated.

It is worth noting that two proteins involved in cell adhesion, identified as serine-aspartate repeat-containing protein D precursor and putative surface protein SAV2496/SAV2497 precursor, were down regulated in response to the treatments with higher levels of NaCl (B, E and F). Bifunctional autolysin was the only protein that displayed an up-regulation in its abundance across all the experimental regimens compared with control samples. The abundance of staphylococcal secretory antigen ssaA2 was increased under more acidic conditions at 35 °C with or without the addition of NaCl (groups C and E), but remained consistent under neutral or alkaline pH (B, D and F). The catalase was significantly up regulated under more alkali conditions (groups D and F), but remained at control levels under more acidic or normal pH (groups B, C and E). These results established that modifying the medium from acidic to alkaline pH reversed the cells response for maintaining ssaA2 and the catalase levels in the cytoplasm. Alkaline shock protein, which is a general stress protein, was only upregulated in experimental group (E), and no changes were observed in any other experimental groups.

Ten out of the 12 proteins that were altered in the bacterial cells exposed to an additional 2.5% NaCl at pH 7 and 37 °C (B) displayed an up-regulation compared with the controls (A) and were all associated with protein synthesis. The two proteins that did not alter included the elongation factor P and proline tRNA ligase. The addition of 5% NaCl at 35 °C at pH 8 (F) resulted in the elevated levels of all these proteins compared with the control. By contrast, however, the addition of the 5% added NaCl at 35 °C and pH 6 (E) resulted in diminished levels of eight out of 12 of this group of proteins, where valine tRNA ligase, aspartyl/glutamyl tRNA (Asn/Gln) amidotransferase subunit B and isoleucine tRNA ligase did not alter in concentration, but proline tRNA ligase was up regulated in concentration compared with the control. When the bacterial cells were subjected to the 35 °C regimes without added NaCl, no general responses were observed for the majority group of the proteins involved in the protein synthesis, but at pH 8 and 35 °C (D), a relative up-regulation was observed in glutamyl tRNA (Gln) amidotransferase subunit A.

**Figure 1:**
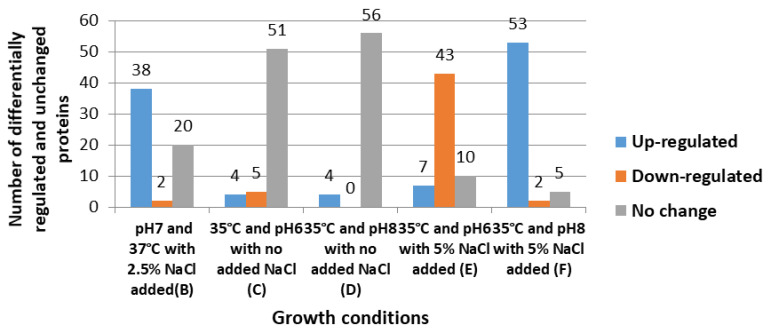
Bacterial proteins differentially regulated or unchanged in each experimental group (**B**–**F**) in comparison to the reference control samples (**A**).

The number of proteins differentially regulated in each experimental group varied according to the growth conditions applied (Figure 1). For example, the cells exposed to pH 8 at 35 °C with an additional 5% NaCl (F) yielded the greatest number of altered proteins (55), including 53 up regulated proteins and two down regulated proteins, whilst the expression of five proteins remained unchanged (Figure 1). The equivalent cultures grown at pH 6 and 35 °C with 5% NaCl (E) showed that the regulation of 50 proteins was altered, including 43 down regulated and seven up regulated, while the expression of 10 proteins remained unchanged. Compared with the reference controls (A), the cells grown at pH 7 and 37 °C with 2.5% NaCl (B) showed 38 up regulated and two down regulated proteins, and the expression of 20 proteins did not change. However, the bacterial cells grown in cultures without NaCl showed few changes in cytoplasmic compositions; the cells exposed to pH 6 and 35 °C (C) had four proteins up regulated and five down regulated, whereas those exposed to pH 8 and 35 °C (D) had only four proteins up regulated and none down regulated.

**Figure 2:**
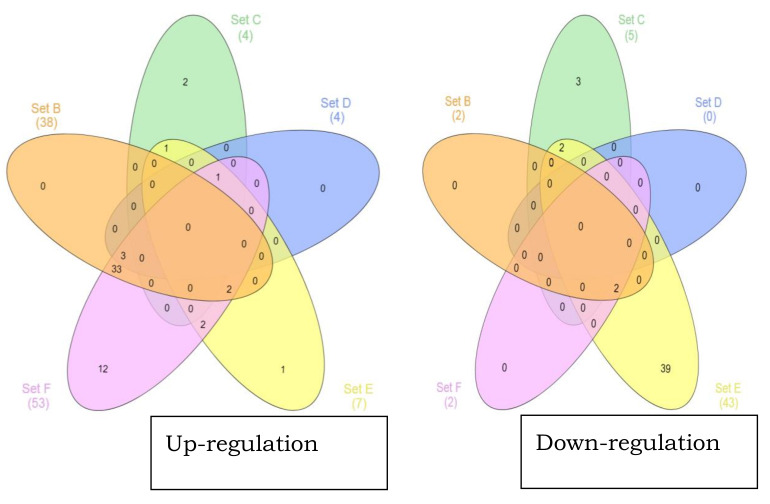
Venn diagram of the differentially regulated proteins. The sum of the number of non-overlapping circles represents the total number of differentially regulated proteins found only in that respective treatment; the overlapping parts of the circles indicate the differentially regulated proteins common between the experimental groups.

Figure 2 shows the distribution of the differentially regulated proteins between the experimental groups. Twelve out of 53 proteins were exclusively up regulated in the experimental group F (pH 8 and 35 °C with additional 5% NaCl), while the remaining proteins were common with other experimental groups (these proteins are listed in Table 1). Group B (pH 7 and 37 °C with 2.5% NaCl), which only differed from the control by having an additional 2.5% NaCl, had the most up regulated proteins (38), but did not show any production of unique proteins. Most of the up regulated proteins in group B were shared with the experimental group (F) (33) and five proteins with other experimental groups (C, D and E). Interestingly, experimental group C had two proteins exclusively up regulated, even though this group only had a total of four up regulated proteins. Group E had one protein uniquely up regulated. Group E (pH 6 and 35 °C and additional 5% NaCl) had the highest number of exclusively down regulated proteins (39 out of 43), followed by group C, which had three proteins uniquely down regulated. Other experimental groups (B, D, and F) did not have any proteins exclusively down regulated.

**Figure 3:**
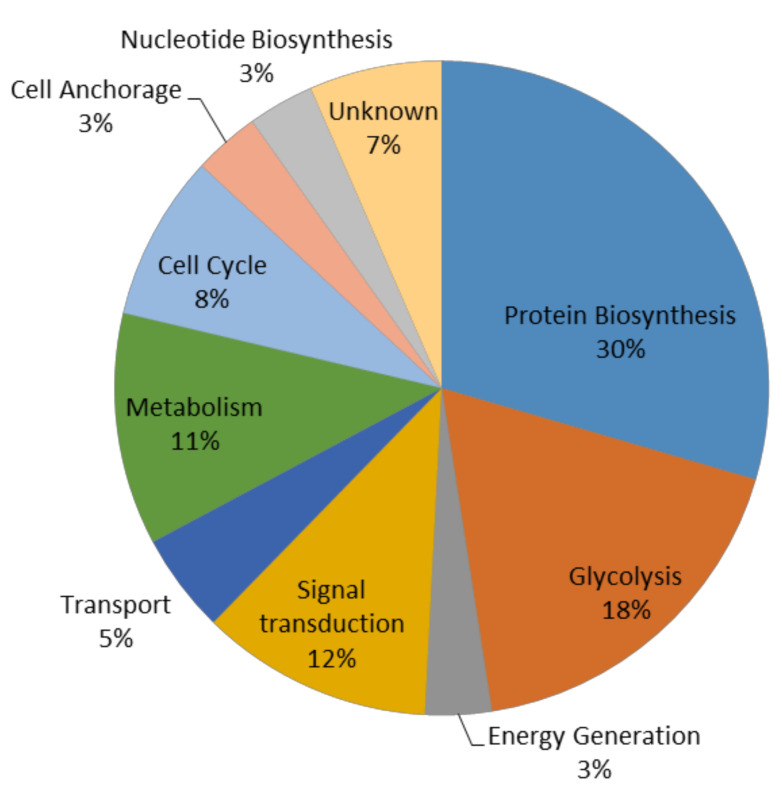
Gene ontology enrichment analysis of the molecular function of the differentially regulated proteins.

Gene ontology functional enrichment analysis by the PANTHER classification system was applied to determine the putative function of 60 proteins that showed significant differences in their abundances (Figure 3). These proteins were classified into 10 functional categories: protein biosynthesis (30%), glycolysis (18%), signal transduction (12%), metabolism (11%), cell cycle (8%), transport (5%), energy generation (3%), cell anchorage (3%), nucleotide biosynthesis (3%), and unknown (7%).

### 3.2. Clustering Analysis

**Figure 4:**
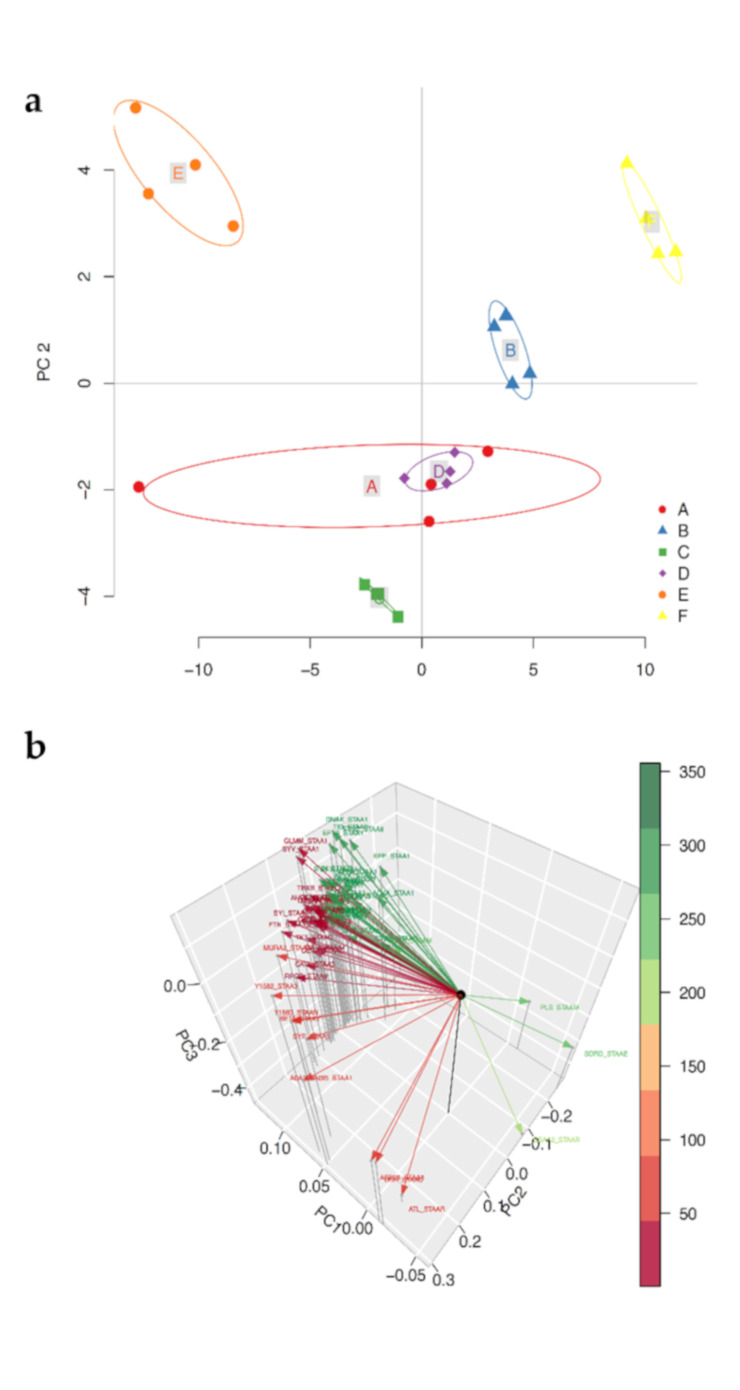
(**a**) PCA analysis of the differentially regulated proteins’ (A) control cultures grown under ideal conditions at pH 7 and 37 °C with no added NaCl; (B) the centroid cultures grown at pH 7 and 37 °C with additional 2.5% NaCl, and the four experimental groups: (C) 35 °C and pH 6 with no added NaCl; (D) 35 °C and pH 8 with no added NaCl; (E) 35 °C and pH 6 with 5% NaCl added; (F) 35 °C and pH 8 with 5% NaCl. (**b**) The loadings PC1, PC2, and PC3 demonstrated the contributions of the proteins to the model.

The cytoplasmic protein data from the cells grown under the reference control (A) and experimental groups (B−F) were further evaluated, using principal component analysis (PCA) for multivariate analysis to find the cytoplasmic protein profile variances between each treatment (Figure 4a). The PCA created a two-component model (R2 = 0.7302 and Q2 = 0.6952181) representing solid significance. The assembling of scores showed that the first component contained the experimentally instilled factors, whereas the second component was modelled within factor variation (Figure 4A). The PCA loadings showed the contribution of the main differential proteins to clustering separation (Figure 4b). The analysis showed that the biological replicates were closely clustered representing great similarity between replicates with characteristic protein profiles between treatments. Furthermore, the PCA displayed that low temperature (35 °C) combined with elevated NaCl (5%) and lower or higher pH (6 or 8) (E and F) were the most significantly distanced groups from the reference control, indicating that the protein profiles were quite different in both treatments compared with the control samples. However, the cultures that did not have NaCl added (C and D) had a similar pattern to the reference control as indicated by less separation from the reference control (A).

**Figure 5:**
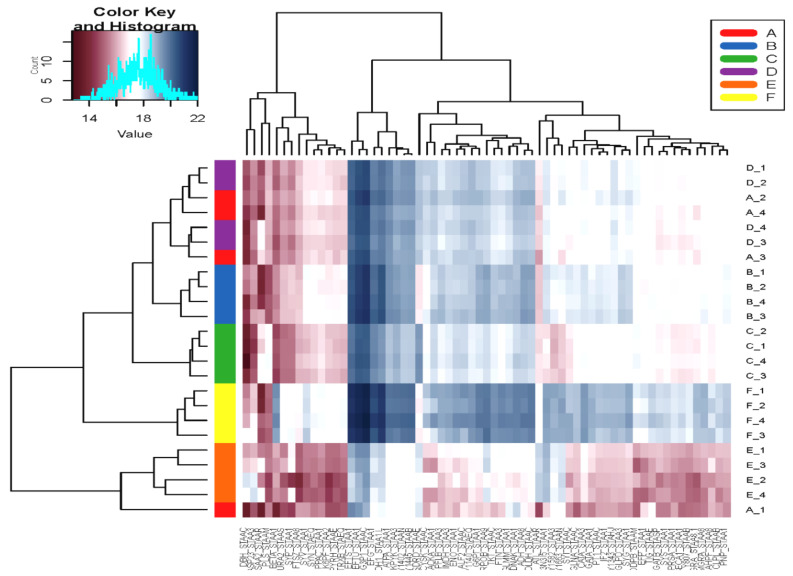
Heatmap represents the abundance of altered proteins extracted from the cells harvested at mid-exponential phase, grown under normal conditions (reference control, **A**) compared to the five different sets of experimental conditions (**B**−**F**). The key color indicates the protein abundance.

The pattern of the global proteins was investigated using hierarchical clustering and a correlation heatmap (Figure 5), which the visualized the protein abundance for all the experimental groups. The bacterial cells grown at 5% NaCl (E and F) showed a very different cluster from those grown under ideal conditions (A). However, the experimental groups (C and D) cultured without NaCl had a pattern more similar to that of the reference control (A) and centroid (B) (Figure 4).

### 3.3. Secreted Protein Results

**Figure 6:**
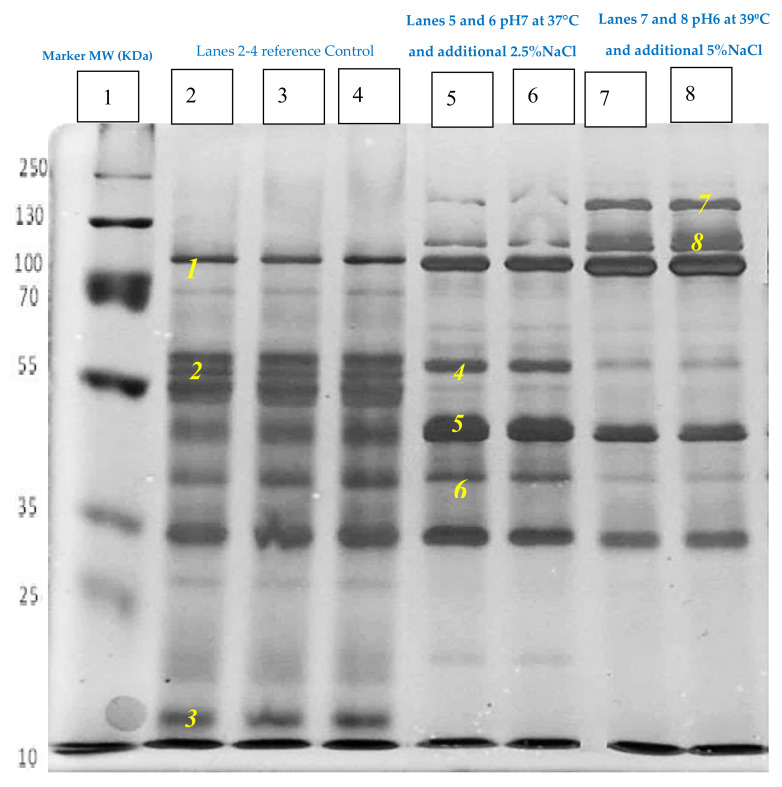
SDS-PAGE analysis of the stationary phase secreted proteins of *S. aureus* grown under the control conditions, compared with the two different experimental treatments. The numbers indicate the bands that were sliced for the identification by LC–MS/MS.

The aim for the final section of the investigation was to monitor the exoproteome responses of *S. aureus* to the changes in the environmental conditions which might be found in the human wound site conditions. *S. aureus* was grown to the stationary phase of growth under control conditions, centroid (additional 2.5% NaCl, pH 7, 37 °C) or with the addition of 5% NaCl at 39 °C and pH 6 representing the environmental parameters existing on the human skin or within a wound site where the temperatures can range from 37 °C–39 °C, pH 6–7, and NaCl can be elevated above the plasma levels. The SDS-PAGE analysis showed that *S. aureus* had secreted a number of proteins by the time the cells reached the stationary phase, but the profiles of the secreted proteins and the intensity of the bands varied between the growth regimes as shown in Figure 6. The gels were repeated for each of the replicates and consistent banding patterns were observed for both the reference controls and treatment samples. A selection of key protein bands is labeled as 1–8 in Figure 6 on the basis of where the bands appeared, in order to represent the changes in intensity for the differences in responses to growth under different conditions.

The analysis revealed that a protein band (labelled *3*) which was approximately 20 KDa was seen very clearly in the cells grown under control conditions, but was absent in the cells grown in altered environmental conditions (Figure 6). This band was identified as the alkyl hydroperoxide reductase subunit. Conversely, the protein bands of approximately 142 and 137 KDa (labeled 7 and 8) were strongly apparent in the treatment samples, but were missing in the control samples (Figure 6, bands 7 and 8). These bands were mainly identified as bifunctional autolysin precursor (Atl). The secretion of these proteins appeared more intense from the cells grown with the additional 5% NaCl at pH6 and at 39 °C in comparison to the cell grown with addition of 2.5% NaCl at pH 7 and at 37 °C. The findings of this study confirm that the Atl protein, which is the major autolysin produced by *S. aureus*, is mostly processed into smaller fragments when this bacterium is grown in TSB with no added NaCl. The addition of salt appeared to increase the production and secretion of Atl. However, the processing of Atl seems to be inhibited with an increasing salt concentration. At 2.5% NaCl, two intermediate lengths of Atl are the predominant fragments (Figure 6, bands no. 4 and 5) with some unprocessed full-length Atl. At 5% NaCl, it seems that most of the Atl being produced was secreted unprocessed as large proteins (Figure 6, bands no. 7 and 8).

Protein bands labeled with 4 and 6 were evidently present in both the control samples and the cells treated with the additional 2.5% NaCl, but were very faint in the cells grown with the additional 5% NaCl at pH6 and at 39 °C. These bands were identified as bifunctional autolysin precursor, lipase 2 precursor, and immunodominant staphylococcal antigen A precursor (Table 2). Moreover, the protein band labelled 1 was relatively less intense in the cells grown under normal conditions compared with the equivalent bands in the treated cells. The identities of the proteins in this band were lipases and autolysin. By contrast, it was apparent that the treatment samples consistently had an absent band at approximately 50 KDa compared with the corresponding band (labeled *2* in the reference control samples). This band contained 30S ribosomal protein S1, catalase, elongation factor Tu, enolase, formate-tetrahydrofolate ligase and ATP synthase subunit beta. The exoproteome results provided evidence that *S. aureus* adjusted its secreted proteins after being exposed to changes in the environmental conditions.

## 4. Discussion

Proteomic approaches were used in this study to investigate the responses of the cytoplasmic and secreted proteins of *S. aureus* to a range of alterations in environmental conditions representative of the human wound site. This study exhibited that *S. aureus* rapidly adapted to the alterations in environmental conditions by inducing significant changes in the proteome compositions. The results provided evidence that there were synergistic interactions between the environmental factors such as temperature and pH, which would differentially manipulate how the bacterial cells would respond to alterations in any specific variable. For instance, the cells responded to an elevated NaCl at pH 6 by down regulating the abundances of cytoplasmic proteins, whereas at pH 8 there was a significant increase in the cytoplasmic proteins compared with the cells grown under normal conditions. These results supported the hypothesis that *S. aureus* adjusted rapidly to the changes in environmental conditions by altering its cytoplasmic proteome and ultimately releasing proteins into the external environments to obtain the optimum homeostasis.

These changes in protein composition could be argued to reflect distinct phenotypes under each experimental group, as previous research revealed that *S. aureus* could form distinct phenotypes after being exposed to identical growth conditions as in the current study [21]. Earlier studies have also demonstrated that colonies of staphylococcal species could grow as SCVs in response to alterations in temperature, osmotic pressure, and pH [8,9]. On this premise, a hypothesis was proposed that bacteria are persistently adapting to modifications in the environment by forming characteristic phenotypes to combat changes in environmental conditions. These significant changes within cytoplasmic and secreted proteins in the current study were interpreted as comprising a necessary component of the cell adaptation processes to enable the cell to consistently adapt to changes in the environment. Similar responses of ribosomal proteins were observed when *S. aureus* was grown under identical experimental design as in the current study [6]. This concept was also demonstrated in *S. lugdunensis* under a similar design, which revealed responses attributable to fatty acids and cell size that were characteristic of the prevailing environmental conditions [19].

The analysis of cytoplasmic proteins in the present study revealed that the major focus of adaptation to environmental challenges involved adjustments in those proteins associated with protein synthesis, which accounted for 30% of the total differentially regulated proteins. The majority of these proteins were altered following the exposure to any of the growth regimes which included the addition of 2.5% or 5% NaCl. The responses by *S. aureus* to growth under additional NaCl at pH 7 or 8 involved the increase in most of the proteins related to protein synthesis, whereas the response at pH 6 involved a down-regulation. It was interesting to note that at pH 6, and at 35 °C with 5% NaCl added (E), the proline tRNA ligase was up regulated when all other proteins associated with protein synthesis were either decreased or showed no significant change. These results suggested that this protein may fulfill an additional/alternative role to protein synthesis required for enabling growth and regulating homeostasis as shown by their independent responses under different environmental challenges.

An earlier study indicated that *S. aureus* responded to prolonged cold stress by up regulating a high number of proteins associated with proteins synthesis [7]. A similar response has been noted in the SCVs of *S. aureus* in response to the antibiotics [32]. This finding is also consistent with a prior study that analyzed the ribosomal proteins of *S. aureus* in the identical sets of growth conditions [6]. Most ribosomal proteins were up regulated in response to a higher NaCl concentration at a normal or more alkaline pH, but an adverse response was recorded when the bacterium was exposed to an additional 5% NaCl at pH 6. A recent study showed significant alterations in the protein compositions of *S. aureus* in response to 10% and 20% NaCl [33]. The authors proposed that in response to multiple environmental stimuli, the cells adapt as required to facilitate survival. The distribution of differentially regulated proteins in the current study was dependent on the set of growth conditions, even though some growth conditions revealed few changes in cytoplasmic proteins, but *S. aureus* had some exclusively regulated proteins in response to these conditions. These results provide strong evidence that protein synthesis is a vital component of the adaptive response by *S. aureus* to alterations in environmental conditions.

The results from this study have shown that the adaptive processes require extensive changes in the secretion of proteins shown by the differential secretions observed between the control and treated samples. The SDS-PAGE analysis of stationary phase secreted proteins of *S. aureus* showed that even with small alterations in the growth conditions implemented by the addition of 2.5% NaCl, differences in both the intensity and the distribution of protein bands were evident from the secretome. When bacterial cells were exposed to more intense changes in environmental conditions by altering temperature, osmolality, and pH, certain substantial changes were noted in the secretome profiles. This result suggests that the bacterium had to pump different proteins into the external media as part of the adaptation process, but it is not yet clear how these proteins would contribute to survival. The analysis indicated that control samples, with no extra NaCl, Atl was processed to various smaller fragments and that was why there was no accumulation of the large Atl and only maybe a little of the intermediate forms of the Atl. However, with the addition of 2.5% NaCl, the Atl was initially produced as an approximately 137 kDa protein, and was then processed into several smaller forms. The smaller subunits seen in bands labelled with *4* and *5* are the two fragments that have originated from the larger Atl due to processing. At 5% NaCl, it appears that the Atl processing was mostly inhibited and almost all of the Atl was present as large proteins. Atl at spot 7 may have the signal peptide and in spot 8 the signal peptide may be missing. Atl protein plays crucial roles in the pathogenicity of *S. aureus* as it is directly mediated in the biofilm development and the secretion of the cytoplasmic proteins from the staphylococcal cell wall [34,35,36]. *S. aureus* had an increased autolytic activity in response to higher NaCl [34].

The secretion of the different proteins or different quantities of proteins could suggest that *S. aureus* has a range of adaptation strategies for survival under various sets of environmental conditions. The elevated intensities of a certain number of proteins compared with the constant secretion of some proteins suggested that these proteins may play specific roles in the adaptation of this bacterium to the applied conditions. In earlier work, *S. aureus* exhibited different secretome profiles in response to silver ions [3]. It is reasonable to hypothesize that these proteins may also have a role in neutralizing the surrounding environments.

A significant reduction in the secretion of enolase and immunodominant antigen staphylococcal A was noted following the exposure to environmental challenges. It has been shown that proteins involved in pathogenicity and metabolism were substantially decreased in response to silver ions [3] suggesting that the bacterium down regulated its metabolism and virulence factors to focus on surviving harsher conditions under nutrient limitations with accumulated waste products. The secretion of lipase 1 and 2 in the current study was more by the cells growing with the addition of NaCl compared with the cells grown without salt. *S. aureus* reduced its ability to form biofilm in the absence of gene-encoded lipase, suggesting that this enzyme was essential for survival and pathogenicity [37,38,39]. The same reduction was noted in *S. aureus* SCVs. Biofilm production is a survival mechanism used by staphylococcal species in response to environmental challenges [40,41].

In the present study, the ribosomal protein S1 was found among the secreted proteins. The 50S ribosomal protein L25, a general stress protein, was secreted together with other proteins after the exposure of *S. aureus* to antimicrobial silver ions [3]. A number of ribosomal proteins have also been found among surface and secreted proteins [42,43], but the functions of these secreted ribosomal proteins remain unclear. Therefore, certain members of the suite of ribosomal proteins may be secreted to the cell surface or into the external environment as a defensive mechanism in response to external challenges from the host’s immune system, antibiotics, and changing environmental conditions. The release of ribosomal proteins may be the result of the increased production of these proteins in the cytoplasm. The exposure of *S. aureus* to extreme cold stress and alkaline conditions combined with elevated osmotic stress led to the significant up-regulation of a high number of ribosomal proteins [6,7]. These proteins may involve in the adaptation processes of *S. aureus* to the prolonged exposure to 4 °C or alkaline conditions combined with elevated osmotic stress.

## 5. Conclusions

The current study indicated that subtle alterations in environmental factors led to significant changes in bacterial cytoplasmic and secreted protein patterns. It showed that the majority of altered cytoplasmic proteins were involved in protein biosynthesis. The analysis of the secreted proteins indicated that fewer proteins were released into external media in response to the changes to environmental conditions. The key finding of the secreted protein analysis was that the Atl protein was released to two fragments under control samples, but this protein was produced at full length and at its two domains. These changes in proteomic profiles would provide the basis for the acclimation mechanisms that have controlled the evolutionary survival of *S. aureus* allowing it to proliferate even in a rapidly changing environment to take the benefit of dominant environments and infectious chances.

## Figures and Tables

**Table 1:** Bacterial proteins differentially regulated following the exposure to changing environmental conditions (B–E) in comparison to the reference control samples (A).

Biological Process	Molecular Function	Uniprot Entry Name	Proteins ID	Experimental Conditions				
				B	C	D	E	F
				37 °C 2.5%NaCl pH7; Centroid	35 °C 0%NaCl pH6;	35 C 0%NaCl pH8;	35 °C 5%NaCl pH6;	35 °C 5%NaCl pH8;
Protein biosynthesis	Elongation factor	EFG_STAA1	Elongation factor G	Up regulated	NC	NC	Down regulated	Up regulated
Protein biosynthesis	Elongation factor	EFTS_STAA1	Elongation factor Ts	Up regulated	NC	NC	Down regulated	Up regulated
Protein biosynthesis	Elongation factor	EFTU_STAA1	Elongation factor Tu	Up regulated	NC	NC	Down regulated	Up regulated
Protein biosynthesis	Elongation factor	EFP_STAA1	Elongation factor P	NC	NC	NC	Down regulated	Up regulated
Protein biosynthesis	Aminoacyl–tRNA synthetase and ligase	SYV_STAA1	Valine__tRNA ligase	Up regulated	NC	NC	NC	Up regulated
Protein biosynthesis	Initiation factor	IF2_STAA1	IF2_STAA1 translation initiation factor IF–2	Up regulated	NC	NC	Down regulated	Up regulated
Protein biosynthesis	Ligase	GATA_STAA2	Glutamyl_tRNA (Gln) amidotransferase subunit A	Up regulated	NC	Up regulated	Down regulated	Up regulated
Protein biosynthesis	Ligase	GATB_STASP	Aspartyl/glutamyl_tRNA (Asn/Gln) amidotransferase subunit B	Up regulated	NC	NC	NC	Up regulated
Protein biosynthesis	Aminoacyl–tRNA synthetase and ligase	SYI1_STAAU	Isoleucine__tRNA ligase	Up regulated	NC	NC	NC	Up regulated d
Protein biosynthesis	Aminoacyl–tRNA synthetase, ligase	SYP_STAA1	Proline__tRNA ligase	NC	NC	NC	Up regulated	Up regulated
Protein biosynthesis	Glycine–tRNA ligase	SYG_STAA1	Glycine__tRNA ligase	Up regulated	NC	NC	Down regulated	Up regulated
Protein biosynthesis	Aminoacyl–tRNA synthetase and ligase	SYS_STAA1	Serine__tRNA ligase	Up regulated	NC	NC	Down regulated	Up regulated
Glycolysis	Oxidoreductase	G3P1_STAAC	Glyceraldehyde_3_phosphate dehydrogenase 1	Up regulated	NC	NC	Down regulated	Up regulated
Glycolysis	Kinase and transferase	KPYK_STAA3	Pyruvate kinase	Up regulated	NC	NC	Down regulated	Up regulated
Glycolysis	Oxidoreductase	DLDH_STAAC	Dihydrolipoyl dehydrogenase	Up regulated	NC	NC	Down regulated	Up regulated
Glycolysis	Oxidoreductase	ODPB_STAAM	Pyruvate dehydrogenase E1 component subunit beta	NC	NC	NC	Down regulated	NC
Glycolysis	Lyase	ALF2_STAAC	Fructose_bisphosphate aldolase	NC	NC	NC	Down regulated	Up regulated
Glycolysis	Allosteric enzyme, kinase and transferase	K6PF_STAA2	Dependent 6_phosphofructokinase	Up regulated	NC	NC	Down regulated	Up regulated
Gluconeogenesis and glycolysis	Isomerase	G6PI_STAA1	Glucose_6_phosphate isomerase	Up regulated	NC	NC	Down regulated	Up regulated
Glycolysis and virulence	Lyase	ENO_STAA1	Enolase	NC	NC	NC	Down regulated	Up regulated
Virulence	Chaperon	CLPL_STAA3	ATP_dependent Clp protease ATP_binding subunit ClpL	NC	NC	NC	Down regulated	Up regulated
Transcription regulation and virulence	Activator, DNA-binding and repressor	MGRA_STAA8	HTH_type transcriptional regulator MgrA	Up regulated	NC	NC	NC	Up regulated
Transcription	Nucleotidyltransferase and transferase	RPOB_STAA9	DNA directed RNA polymerase subunit beta	Up regulated	NC	NC	NC	Up regulated
Stress response	Oxidoreductase	LDH1_STAA1	L_lactate dehydrogenase 1	Up regulated	NC	NC	Down regulated	Up regulated
Stress response	Chaperone	DNAK_STAA1	Chaperone protein DnaK	NC	Down regulated	NC	Down regulated	Up regulated
General stress protein	Alkaline pH tolerance	ASP23_STAA3	Alkaline shock protein 23	NC	NC	NC	Up regulated	NC
DNA damage repair and stress response	Hydrolase	HCHA_STAAW	Protein/nucleic acid deglycase HchA	Up regulated	Down regulated	NC	Up regulated	Up regulated
ATP synthesis, hydrogen ion transport, ion transport, transport	Translocase	ATPA_STAA1	ATP synthase subunit alpha	NC	Down regulated	NC	Down regulated	Up regulated
Ion storage	Oxidoreductase	FTN_STAA3	Ferritin	Up regulated	NC	NC	NC	Up regulated
**Alcohol metabolic process**	Oxidoreductase	ADH_STAA3	Alcohol dehydrogenase	Up regulated	NC	NC	Down regulated	Up regulated
Hydrogen peroxide catabolic process a	Oxidoreductase and peroxidase	CATA_STAA3	Catalase;	Up regulated	NC	Up regulated	NC	Up regulated
DNA recombination	Transferase	TKT_STAAC	Transketolase	Up regulated	NC	NC	Down regulated	Up regulated
Glycine betaine biosynthetic process from choline	Oxidoreductase	BETA_STAA1	Choline dehydrogenase	NC	NC	NC	NC	Up regulated
mRNA catabolic process and RNA processing	Nucleotidyltransferase RNA-binding and transferase	PNP_STAA1	Polyribonucleotide nucleotidyltransferase	Up regulated	NC	NC	Down regulated	Up regulated
Glycerol metabolism	Oxidoreductase	GLPD_STAA3	Aerobic glycerol_3_phosphate dehydrogenase	Up regulated	NC	NC	Down regulated	Up regulated
GMP biosynthesis	Oxidoreductase	IMDH_STAA3	Inosine_5__monophosphate dehydrogenase	Up regulated	NC	NC	Down regulated	Up regulated
Carbohydrate metabolism	Acyltransferase	PFLB_STAA3	Formate acetyltransferase	Up regulated	NC	NC	Down regulated	Up regulated
Carbohydrate metabolic process	Magnesium ion binding and phosphoglucosamine mutase activity	GLMM_STAA1	Phosphoglucosamine mutase	Up regulated	NC	NC	Down regulated	Up regulated
Acetyl-CoA biosynthetic process and organic acid metabolic process	Kinase transferase	ACKA_STAA1	Acetate kinase	NC	NC	NC	Down regulated	NC
Protein transport, translocation and transport	ATPase-coupled protein transmembrane transporter activity, ATP binding and metal ion binding	SECA1_STAA1	Protein translocase subunit SecA 1	Up regulated	NC	NC	Down regulated	Up regulated
Uncharacterized	Uncharacterized	Y1663_STAAN	UPF0342 protein SA1663	NC	Down regulated	NC	Up regulated	Up regulated
Uncharacterized	Uncharacterized	Y1402_STAAN	UPF0365 protein SA1402	NC	NC	NC	Down regulated	Up regulated
Uncharacterized	Uncharacterized	Y1582_STAA3	UPF0337 protein SAUSA300_1582	Up regulated	Down regulated	NC	Up regulated	Up regulated
Amino acid biosynthesis	Transferase	CYSK_STAAC	CYSK_STAAC cysteine synthase	NC	NC	NC	Down regulated	NC
Amino acid biosynthesis	Ligase	GLNA_STAAC	GLNA_STAAC glutamine synthetase	Up regulated	NC	NC	Down regulated	Up regulated
Cell wall biogenesis/degradation	Hydrolase	ATL_STAAR	Bifunctional autolysin precursor	Up regulated	Up regulated	Up regulated	Up regulated	Up regulated
Cell wall biosynthesis	Adhesion	SDRD_STAAE	Serine_aspartate repeat_containing protein D	Down regulated	Up regulated	NC	Down regulated	Down regulated
Cell redox homeostasis and response to reactive oxygen species	Oxidoreductase	AHPF_STAA8	Alkyl hydroperoxide reductase subunit F	NC	NC	NC	NC	Up regulated
Energy production and conversion	Oxidoreductase	Y807_STAAB	NADH dehydrogenase_like protein SAB0807	Up regulated	NC	NC	Down regulated	Up regulated
Energy production and conversion	Hydrolase	PPAC_STAA1	Probable manganese_dependent inorganic pyrophosphatase	Up regulated	NC	Up regulated	Down regulated	Up regulated
Uncharacterized	Uncharacterized	PLS_STAAM	Putative surface protein SAV2496/SAV2497; Flags: Precursor	Down regulated	Up regulated	NC	Down regulated	Down regulated
Asparaginyl–tRNA aminoacylation	Asparagine–tRNA ligase activity, ATP binding and nucleic acid binding	SYN_LISIN	Asparagine__tRNA ligase	Up regulated	NC	NC	Down regulated	Up regulated
Protein folding	peptidyl-prolyl cis-trans isomerase activity	PRSA_STAA1	Foldase protein PrsA	NC	NC	NC	Down regulated	Up regulated
Cell cycle, cell division, and Septation	GTPase activity and GTP binding	FTSZ_STAA8	Cell division protein FtsZ	NC	NC	NC	Down regulated	Up regulated
Cell cycle and cell division	Chaperone, isomerase and rotamase	TIG_STAA1	Trigger factor	NC	NC	NC	Down regulated	Up regulated
Cell cycle, cell division, cell shape, cell wall biogenesis/degradation, peptidoglycan synthesis	Transferase	MURA2_STAAN	UDP_N_acetylglucosamine 1_carboxyvinyltransferase 2	Up regulated	NC	NC	NC	Up regulated
Virulence	Hydrolase	SSAA2_STAA8	Staphylococcal secretory antigen ssaA2	NC	Up regulated	NC	Up regulated	NC
Removal of superoxide radicals	Oxidoreductase	TRXB_STAEQ	Thioredoxin reductase	Up regulated	NC	NC	Down regulated	Up regulated
Phosphotransferase system, sugar transport and transport	Kinase and transferase	PT1_STAAC	Phosphoenolpyruvate_protein phosphotransferase	Up regulated	NC	NC	Down regulated	Up regulated
Pyrimidine biosynthesis	Allosteric enzyme, kinase and transferase	PYRH_STAAE	Uridylate kinase	Up regulated	NC	NC	Down regulated	Up regulated

**Table 2:** Identified proteins within the bands that varied in abundance between the strains and treatments.

Band #	Accession	Protein	Scores	MW (kDa)	Peptides
1	LIP1_STAAC	Lipase 1	1675.5	76.6	28
	LIP2_STAAM	Lipase 2 precursor	1592.0	76.5	25
	tr|F0D7U9|F0D7U9_STAAU	Autolysin	810.9	137.0	15
	**Accession**	**Protein**	**Scores**	**MW (kDa)**	**Peptides**
2	CATA_STAAB	Catalase	710.7	58.3	28
	RS1_STAAC	30S ribosomal protein S1	615.1	43.3	9
	H0AR71_STAAU	Elongation factor Tu	431.5	43.1	20
	tr|T1Y7Q4|T1Y7Q4_STAAU	Enolase	406.0	47.0	8
	tr|T1YAV3|T1YAV3_STAAU	Formate-tetrahydrofolate ligase	349.2	61.1	8
	tr|N5DN28|N5DN28_STAAU	ATP synthase subunit beta	309.1	51.4	6
3	**Accession**	**Protein**	**Scores**	**MW (kDa)**	**Peptides**
	tr|S9RPT3|S9RPT3_STAAU	Alkyl hydroperoxide reductase subunit C	518.2	20.9	7
4	**Accession**	**Protein**	**Scores**	**MW (kDa)**	**Peptides**
	ATL_STAAM	ATL_STAAM Bifunctional autolysin precursor (includes: N-acetylmuramoyl-L-alanine amidase (EC 3.5.1.28); mannosyl-glycoprotein endo-beta-N-acetylglucosaminidase (EC 3.2.1.96))	3684.0	136.7	47
5	**Accession**	**Protein**	**Scores**	**MW (kDa)**	**Peptides**
	ATL_STAAM	Bifunctional autolysin precursor (includes: N-acetylmuramoyl-L-alanine amidase (EC 3.5.1.28); mannosyl-glycoprotein endo-beta-N-acetylglucosaminidase (EC 3.2.1.96)	3684.2	136.7	57
	LTAS_STAAC	Glycerol phosphate lipoteichoic acid synthase	1940.1	74.4	32
	tr|F3TIS4|F3TIS4_STAAU	Triacylglycerol lipase	336.5	72.2	6
	tr|G7ZSU9|G7ZSU9_STAAU	Probable transglycosylase isaA	607.7	24.1	7
6	**Accession**	**Protein**	**Scores**	**MW (kDa)**	**Peptides**
	LIP2_STAAC	Lipase 2 precursor (EC 3.1.1.3)	620.7	76.3	12
	ISAA_STAAC	Immunodominant staphylococcal antigen A precursor	441.1	24.2	5
7	**Accession**	**Protein**	**Scores**	**MW (kDa)**	**Peptides**
	ATL_STAAW	Bifunctional autolysin precursor (includes: N-acetylmuramoyl-L-alanine amidase (EC 3.5.1.28); mannosyl-glycoprotein endo-beta-N-acetylglucosaminidase)	5871.0	137.3	88
	SDRD_STAAE	Serine-aspartate repeat-containing protein D	378.3	142.7	6
8	**Accession**	**Protein**	**Scores**	**MW (kDa)**	**Peptides**
	ATL_STAAW	Bifunctional autolysin precursor (includes: N-acetylmuramoyl-L-alanine amidase (EC 3.5.1.28); mannosyl-glycoprotein endo-beta-N-acetylglucosaminidase (EC 3.2.1.96))	4932.6	137.3	77
	LIP2_STAAC	Lipase 2 precursor (EC 3.1.1.3)	404.2	76.3	9

## Supplementary Materials

The following are available online at https://www.mdpi.com/2076-2607/8/7/1082/s1, Table S1: Peptide sequences and relative statistical information; Table S2: The sum of all peptide intensities quantified for each proteins.
